# Hydrazinediium bis­(6-carboxy­pyridazine-3-carboxyl­ate) dihydrate

**DOI:** 10.1107/S1600536808001037

**Published:** 2008-01-16

**Authors:** Wojciech Starosta, Janusz Leciejewicz

**Affiliations:** a Institute of Nuclear Chemistry and Technology, ul. Dorodna 16, 03-195 Warszawa, Poland

## Abstract

The triclinic unit cell of the title compound, N_2_H_6_^2+^·2C_6_H_3_N_2_O_4_^−^·2H_2_O, contains one doubly protonated hydrazine cation which lies on an inversion centre, two symmetry-related singly deprotonated 6-carboxy­pyridazine-3-carboxyl­ate anions and two symmetry-related solvent water mol­ecules. The anions inter­act *via* hydrogen bonds to form double ribbons which are bridged by hydrogen bonds donated by hydrazinediium cations and water molecules.

## Related literature

For the crystal structures of two polymorphs of the hydrazine adduct of pyrazole-3,5-dicarboxylic acid, see Kumar *et al.* (2007 ▶). Singly protonated hydrazine cations and di(aqua-*O*)bis­(pyridazine-3,6-dicarboxyl­ato-*N*,*O*)magnesium(II) anions have also been observed (Gryz *et al.*, 2004 ▶). For related literature, see: Starosta & Leciejewicz (2004 ▶); Sueur *et al.* (1987 ▶).
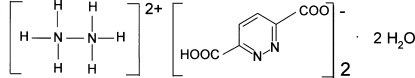


## Experimental

### 

#### Crystal data

N_2_H_6_^2+^·2C_6_H_3_N_2_O_4_^−^·2H_2_O
*M* *_r_* = 404.31
Triclinic, 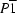

*a* = 5.1727 (10) Å
*b* = 6.6257 (13) Å
*c* = 12.271 (3) Å
α = 102.08 (3)°
β = 93.92 (3)°
γ = 107.44 (3)°
*V* = 388.47 (17) Å^3^
*Z* = 1
Mo *K*α radiation
μ = 0.15 mm^−1^
*T* = 293 (2) K
0.16 × 0.08 × 0.07 mm

#### Data collection

Kuma KM-4 four-circle diffractometer
Absorption correction: none
2512 measured reflections
2279 independent reflections
1431 reflections with *I* > 2σ(*I*)
*R*_int_ = 0.017
3 standard reflections every 200 reflections intensity decay: 3.72%

#### Refinement

*R*[*F* ^2^ > 2σ(*F* ^2^)] = 0.040
*wR*(*F* ^2^) = 0.133
*S* = 1.02
2279 reflections
159 parameters
H atoms treated by a mixture of independent and constrained refinement
Δρ_max_ = 0.52 e Å^−3^
Δρ_min_ = −0.23 e Å^−3^

### 

Data collection: *KM-4 Software* (Kuma, 1996 ▶); cell refinement: *KM-4 Software*; data reduction: *DATAPROC* (Kuma, 2001 ▶); program(s) used to solve structure: *SHELXS97* (Sheldrick, 2008 ▶); program(s) used to refine structure: *SHELXL97* (Sheldrick, 2008 ▶); molecular graphics: *SHELXTL* (Sheldrick, 2008 ▶); software used to prepare material for publication: *SHELXL97*.

## Supplementary Material

Crystal structure: contains datablocks I, global. DOI: 10.1107/S1600536808001037/at2534sup1.cif

Structure factors: contains datablocks I. DOI: 10.1107/S1600536808001037/at2534Isup2.hkl

Additional supplementary materials:  crystallographic information; 3D view; checkCIF report

## Figures and Tables

**Table 1:** Hydrogen-bond geometry (Å, °)

*D*—H⋯*A*	*D*—H	H⋯*A*	*D*⋯*A*	*D*—H⋯*A*
O6—H62⋯N1^i^	0.81 (3)	2.23 (3)	3.031 (2)	174 (3)
N3—H51⋯O4^ii^	0.96 (2)	1.85 (2)	2.770 (2)	160 (2)
O6—H61⋯O4^ii^	0.98 (3)	1.99 (3)	2.9581 (18)	168 (2)
O6—H61⋯N1^ii^	0.98 (3)	2.45 (2)	3.039 (2)	118.5 (18)
N3—H53⋯N2^i^	0.91 (2)	1.95 (2)	2.8287 (18)	163 (2)
N3—H53⋯O2^i^	0.91 (2)	2.50 (2)	3.0627 (19)	120.7 (17)
N3—H52⋯O6^iii^	1.04 (2)	1.71 (2)	2.7473 (18)	176.6 (19)
O1—H1⋯O3^iv^	1.03 (4)	1.51 (4)	2.5152 (16)	165 (3)

Symmetry codes: (i) 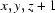; (ii) 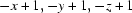; (iii) 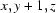; (iv) 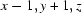.

**Fig. 1.**
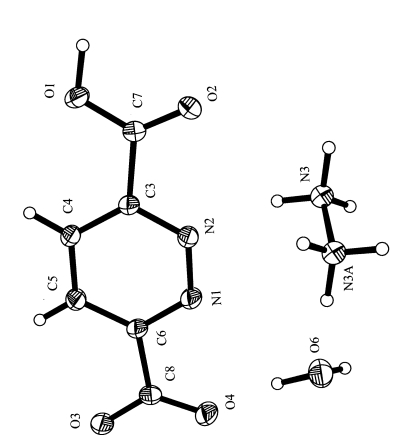
The asymmetric unit with atom labelling scheme and 50% probability displacement ellipsoids. The hydrazine cation is symmetry complete (symmetry code: (A) -x + 1, -y + 1, -z + 1).

**Fig. 2.**
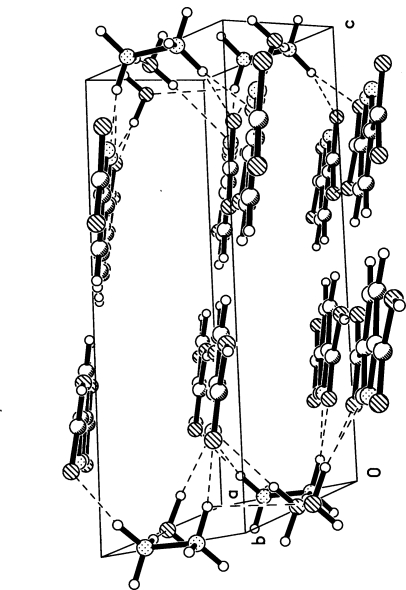
Packing diagram of the structure of (I).

## supplementary crystallographic information

### Comment

The structure of the title compound (I) is composed of one doubly-protonated hydrazine cation having its geometrical centre on an inversion centre at 1/2,0,0, two symmetry related singly-deprotonated pyridazine-3,6-dicarboxylate anions and a pair of symmetry related solvent water molecules. Fig.1 shows the asymetric unit with atom labelling scheme. Atoms forming the pyridazine ring are coplanar (r.m.s.0.057 Å). The carboxylate moiety (C7/O1/O2) makes an angle of 3.8 (1) ° with the pyridazine ring, while the (C8/O3/O4) group makes an angle of 1.1 (1) °. Bond lengths and bond angles within the title anion agree well with those reported in the structures of both modifications of the parent acid (Sueur *et al.*, 1987, Starosta & Leciejewicz, 2004). A fairly strong hydrogen bond of 2.515 (2) Å is observed between the protonated O atom of the carboxylic group acting as a donor and the deprotonated carboxylate O atom in the adjacent anion giving rise to polyionic ribbons composed of pairs of anions (Fig. 2). The ribbons are bridged by weaker bonds in which the hydrazine cations and solvation water molecules are the donors and the anions' O atoms and hetero-ring N atoms act as acceptors.

### Experimental

In the course of experiments aiming to obtain single crystals of a calcium complex with pyridazine-3,6-dicarboxylate ligand, single crystals of either the triclinic modification of the pyridazine-3,6-dicarboxylic acid dihydrate (Starosta & Leciejewicz, 2004) or of the title compound were found in the mass of polycrystalline material. The crystals of the title compound appeared when hydrazine was used to maintain the acidicity of the initial solution.

### Refinement

All H atoms were located in a difference map and refined with isotropic displacement parameters.

### Crystal data

**Table:**

N_2_H_6_^2+^·2C_6_H_3_N_2_O_4_^–^·2H_2_O	*Z* = 1
*M**_r_* = 404.31	*F*_000_ = 210
Triclinic, *P*1	*D*_x_ = 1.728 Mg m^−^^3^
Hall symbol: -P 1	Mo *K*α radiation λ = 0.71073 Å
*a* = 5.1727 (10) Å	Cell parameters from 25 reflections
*b* = 6.6257 (13) Å	θ = 6–15º
*c* = 12.271 (3) Å	µ = 0.15 mm^−^^1^
α = 102.08 (3)º	*T* = 293 (2) K
β = 93.92 (3)º	Rectangular plate, colourless
γ = 107.44 (3)º	0.16 × 0.08 × 0.07 mm
*V* = 388.47 (17) Å^3^	

### Data collection

**Table:**

Kuma KM-4 four-circle diffractometer	*R*_int_ = 0.017
Radiation source: fine-focus sealed tube	θ_max_ = 30.1º
Monochromator: graphite	θ_min_ = 1.7º
*T* = 293(2) K	*h* = −7→0
profile data from ω/2θ scans	*k* = −8→9
Absorption correction: none	*l* = −17→17
2512 measured reflections	3 standard reflections
2279 independent reflections	every 200 reflections
1431 reflections with *I* > 2σ(*I*)	intensity decay: 3.7%

### Refinement

**Table:**

Refinement on *F*^2^	Secondary atom site location: difference Fourier map
Least-squares matrix: full	Hydrogen site location: inferred from neighbouring sites
*R*[*F*^2^ > 2σ(*F*^2^)] = 0.040	H atoms treated by a mixture of independent and constrained refinement
*wR*(*F*^2^) = 0.133	*w* = 1/[σ^2^(*F*_o_^2^) + (0.092*P*)^2^] where *P* = (*F*_o_^2^ + 2*F*_c_^2^)/3
*S* = 1.02	(Δ/σ)_max_ < 0.001
2279 reflections	Δρ_max_ = 0.52 e Å^−^^3^
159 parameters	Δρ_min_ = −0.23 e Å^−^^3^
Primary atom site location: structure-invariant direct methods	Extinction correction: none

### Special details

**Table:**

Geometry. All e.s.d.'s (except the e.s.d. in the dihedral angle between two l.s. planes) are estimated using the full covariance matrix. The cell e.s.d.'s are taken into account individually in the estimation of e.s.d.'s in distances, angles and torsion angles; correlations between e.s.d.'s in cell parameters are only used when they are defined by crystal symmetry. An approximate (isotropic) treatment of cell e.s.d.'s is used for estimating e.s.d.'s involving l.s. planes.
Refinement. Refinement of *F*^2^ against ALL reflections. The weighted *R*-factor *wR* and goodness of fit *S* are based on *F*^2^, conventional *R*-factors *R* are based on *F*, with *F* set to zero for negative *F*^2^. The threshold expression of *F*^2^ > σ(*F*^2^) is used only for calculating *R*-factors(gt) *etc*. and is not relevant to the choice of reflections for refinement. *R*-factors based on *F*^2^ are statistically about twice as large as those based on *F*, and *R*- factors based on ALL data will be even larger.

### Fractional atomic coordinates and isotropic or equivalent isotropic displacement parameters (Å^2^)

**Table:**

	*x*	*y*	*z*	*U*_iso_*/*U*_eq_	
O2	0.0745 (3)	1.1260 (2)	0.19096 (9)	0.0301 (3)	
O3	0.6795 (3)	0.37197 (19)	0.35261 (9)	0.0292 (3)	
O4	0.7130 (3)	0.4033 (2)	0.17502 (10)	0.0319 (3)	
O1	−0.0377 (3)	1.1253 (2)	0.36330 (10)	0.0315 (3)	
N2	0.3236 (3)	0.8346 (2)	0.19854 (10)	0.0248 (3)	
N1	0.4514 (3)	0.6882 (2)	0.19574 (10)	0.0261 (3)	
C6	0.4853 (3)	0.6120 (2)	0.28600 (11)	0.0198 (3)	
C3	0.2223 (3)	0.9032 (2)	0.29085 (11)	0.0191 (3)	
C7	0.0773 (3)	1.0655 (2)	0.27720 (12)	0.0212 (3)	
C4	0.2473 (3)	0.8274 (2)	0.38706 (12)	0.0238 (3)	
C8	0.6396 (3)	0.4472 (2)	0.26852 (12)	0.0215 (3)	
C5	0.3863 (4)	0.6784 (3)	0.38484 (12)	0.0250 (3)	
O6	0.1862 (3)	0.3212 (2)	0.98874 (10)	0.0295 (3)	
N3	0.3528 (3)	0.9661 (2)	0.99354 (11)	0.0233 (3)	
H5	0.169 (4)	0.871 (3)	0.4510 (18)	0.031 (5)*	
H6	0.411 (4)	0.628 (3)	0.4471 (19)	0.037 (6)*	
H62	0.268 (6)	0.416 (5)	1.044 (2)	0.059 (8)*	
H51	0.288 (5)	0.840 (4)	0.9317 (19)	0.038 (6)*	
H61	0.223 (5)	0.395 (4)	0.927 (2)	0.045 (6)*	
H53	0.307 (5)	0.924 (3)	1.0576 (18)	0.035 (5)*	
H52	0.288 (5)	1.098 (4)	0.9885 (18)	0.036 (5)*	
H1	−0.136 (8)	1.234 (6)	0.350 (3)	0.117 (14)*	

### Atomic displacement parameters (Å^2^)

**Table:**

	*U*^11^	*U*^22^	*U*^33^	*U*^12^	*U*^13^	*U*^23^
O2	0.0426 (8)	0.0379 (6)	0.0231 (5)	0.0268 (6)	0.0091 (5)	0.0143 (4)
O3	0.0400 (7)	0.0348 (6)	0.0259 (5)	0.0261 (5)	0.0095 (5)	0.0130 (4)
O4	0.0465 (8)	0.0369 (6)	0.0248 (5)	0.0295 (6)	0.0119 (5)	0.0082 (4)
O1	0.0459 (8)	0.0394 (7)	0.0263 (5)	0.0315 (6)	0.0165 (5)	0.0148 (5)
N2	0.0362 (8)	0.0282 (6)	0.0197 (5)	0.0208 (6)	0.0089 (5)	0.0098 (5)
N1	0.0382 (8)	0.0294 (7)	0.0215 (6)	0.0231 (6)	0.0105 (5)	0.0092 (5)
C6	0.0237 (7)	0.0200 (6)	0.0200 (6)	0.0123 (5)	0.0051 (5)	0.0055 (5)
C3	0.0225 (7)	0.0199 (6)	0.0184 (6)	0.0109 (5)	0.0044 (5)	0.0056 (5)
C7	0.0238 (7)	0.0222 (7)	0.0210 (6)	0.0119 (6)	0.0037 (5)	0.0053 (5)
C4	0.0339 (8)	0.0279 (7)	0.0179 (6)	0.0194 (6)	0.0084 (6)	0.0077 (5)
C8	0.0249 (8)	0.0202 (6)	0.0231 (6)	0.0124 (6)	0.0036 (5)	0.0058 (5)
C5	0.0373 (9)	0.0284 (7)	0.0190 (6)	0.0206 (7)	0.0081 (6)	0.0102 (5)
O6	0.0375 (7)	0.0290 (6)	0.0237 (6)	0.0128 (5)	0.0049 (5)	0.0070 (4)
N3	0.0225 (7)	0.0293 (7)	0.0220 (6)	0.0121 (5)	0.0057 (5)	0.0084 (5)

### Geometric parameters (Å, °)

**Table:**

O2—C7	1.2082 (18)	C3—C7	1.5128 (18)
O3—C8	1.2666 (17)	C4—C5	1.382 (2)
O4—C8	1.2376 (18)	C4—H5	0.94 (2)
O1—C7	1.2956 (18)	C5—H6	0.91 (2)
O1—H1	1.03 (4)	O6—H62	0.81 (3)
N2—N1	1.3241 (17)	O6—H61	0.98 (3)
N2—C3	1.3313 (18)	N3—N3^i^	1.440 (3)
N1—C6	1.3317 (17)	N3—H51	0.96 (2)
C6—C5	1.387 (2)	N3—H53	0.91 (2)
C6—C8	1.5236 (18)	N3—H52	1.04 (2)
C3—C4	1.3889 (19)		
			
C7—O1—H1	111 (2)	C3—C4—H5	122.1 (13)
N1—N2—C3	120.23 (12)	O4—C8—O3	127.45 (13)
N2—N1—C6	120.34 (12)	O4—C8—C6	116.92 (12)
N1—C6—C5	122.08 (13)	O3—C8—C6	115.63 (12)
N1—C6—C8	113.36 (12)	C4—C5—C6	117.66 (13)
C5—C6—C8	124.56 (12)	C4—C5—H6	119.8 (14)
N2—C3—C4	122.16 (13)	C6—C5—H6	122.5 (14)
N2—C3—C7	112.66 (12)	H62—O6—H61	103 (2)
C4—C3—C7	125.17 (13)	N3^i^—N3—H51	106.7 (14)
O2—C7—O1	126.02 (13)	N3^i^—N3—H53	105.3 (14)
O2—C7—C3	119.91 (13)	H51—N3—H53	108 (2)
O1—C7—C3	114.07 (12)	N3^i^—N3—H52	109.5 (13)
C5—C4—C3	117.50 (13)	H51—N3—H52	117.5 (18)
C5—C4—H5	120.3 (13)	H53—N3—H52	109.0 (18)

Symmetry codes: (i) −*x*+1, −*y*+2, −*z*+2.

### Hydrogen-bond geometry (Å, °)

**Table:**

*D*—H···*A*	*D*—H	H···*A*	*D*···*A*	*D*—H···*A*
O6—H62···N1^ii^	0.81 (3)	2.23 (3)	3.031 (2)	174 (3)
N3—H51···O4^iii^	0.96 (2)	1.85 (2)	2.770 (2)	160 (2)
O6—H61···O4^iii^	0.98 (3)	1.99 (3)	2.9581 (18)	168 (2)
O6—H61···N1^iii^	0.98 (3)	2.45 (2)	3.039 (2)	118.5 (18)
N3—H53···N2^ii^	0.91 (2)	1.95 (2)	2.8287 (18)	163 (2)
N3—H53···O2^ii^	0.91 (2)	2.50 (2)	3.0627 (19)	120.7 (17)
N3—H52···O6^iv^	1.04 (2)	1.71 (2)	2.7473 (18)	176.6 (19)
O1—H1···O3^v^	1.03 (4)	1.51 (4)	2.5152 (16)	165 (3)

Symmetry codes: (ii) *x*, *y*, *z*+1; (iii) −*x*+1, −*y*+1, −*z*+1; (iv) *x*, *y*+1, *z*; (v) *x*−1, *y*+1, *z*.
